# Diagnostic Accuracy of Confocal Laser Endomicroscopy for the Diagnosis of Oral Squamous Cell Carcinoma: A Systematic Review and Meta-Analysis

**DOI:** 10.3390/ijerph182312390

**Published:** 2021-11-25

**Authors:** Sneha Sethi, Xiangqun Ju, Richard M. Logan, Paul Sambrook, Robert A. McLaughlin, Lisa M. Jamieson

**Affiliations:** 1 Australian Research Centre for Population Oral Health, Adelaide Dental School, Faculty of Health and Medical Sciences, University of Adelaide, Adelaide, SA 5005, Australia; xiangqun.ju@adelaide.edu.au (X.J.); lisa.jamieson@adelaide.edu.au (L.M.J.); 2 Adelaide Dental School, Faculty of Health and Medical Sciences, University of Adelaide, Adelaide, SA 5005, Australia; richard.logan@adelaide.edu.au (R.M.L.); paul.sambrook@adelaide.edu.au (P.S.); 3 Oral and Maxillofacial Surgery Unit, SA Dental Service, Adelaide, SA 5005, Australia; 4 School of Biomedicine, Faculty of Health and Medical Sciences, University of Adelaide, Adelaide, SA 5005, Australia; robert.mclaughlin@adelaide.edu.au; 5 Australian Research Council Centre of Excellence for Nanoscale Biophotonics, The University of Adelaide, Adelaide, SA 5005, Australia; 6 Institute of Photonics and Advanced Sensing, The University of Adelaide, Adelaide, SA 5005, Australia

**Keywords:** oral squamous cell carcinoma, confocal laser endomicroscopy, systematic review, meta-analysis, diagnostic test accuracy

## Abstract

Background: Advances in treatment approaches for patients with oral squamous cell carcinoma (OSCC) have been unsuccessful in preventing frequent recurrences and distant metastases, leading to a poor prognosis. Early detection and prevention enable an improved 5-year survival and better prognosis. Confocal Laser Endomicroscopy (CLE) is a non-invasive imaging instrument that could enable an earlier diagnosis and possibly help in reducing unnecessary invasive surgical procedures. Objective: To present an up to date systematic review and meta-analysis assessing the diagnostic accuracy of CLE in diagnosing OSCC. Materials and Methods. PubMed, Scopus, and Web of Science databases were explored up to 30 June 2021, to collect articles concerning the diagnosis of OSCC through CLE. Screening: data extraction and appraisal was done by two reviewers. The quality of the methodology followed by the studies included in this review was assessed using the Quality Assessment of Diagnostic Accuracy Studies-2 (QUADAS-2) tool. A random effects model was used for the meta-analysis. Results: Six studies were included, leading to a total number of 361 lesions in 213 patients. The pooled sensitivity and specificity were 95% (95% CI, 92–97%; I^2^ = 77.5%) and 93% (95% CI, 90–95%; I^2^ = 68.6%); the pooled positive likelihood ratios and negative likelihood ratios were 10.85 (95% CI, 5.4–21.7; I^2^ = 55.9%) and 0.08 (95% CI, 0.03–0.2; I^2^ = 83.5%); and the pooled diagnostic odds ratio was 174.45 (95% CI, 34.51–881.69; I^2^ = 73.6%). Although risk of bias and heterogeneity is observed, this study validates that CLE may have a noteworthy clinical influence on the diagnosis of OSCC, through its high sensitivity and specificity. Conclusions: This review indicates an exceptionally high sensitivity and specificity of CLE for diagnosing OSCC. Whilst it is a promising diagnostic instrument, the limited number of existing studies and potential risk of bias of included studies does not allow us to draw firm conclusions. A conclusive inference can be drawn when more studies, possibly with homogeneous methodological approach, are performed.

## Highlights

- Confocal Laser Endomicroscopy (CLE) has very high sensitivity and specificity for diagnosing Oral squamous cell carcinoma (OSCC);
- Transference of the first experimental results of CLE in the oral cavity of humans into an effective and evidence based clinical setting is recommended;
- A conclusive statement can only be made when additional comparable studies with homogeneous methodological strategies will be undertaken.

## 1. Introduction

Head and neck squamous cell carcinoma (HNSCC) is the sixth most prevalent cancer [1] globally, contributing towards 5% of all human malignancies [2]. Incidence and mortality due to HNSCC in 2018 was reported as 890,000 and 450,000, respectively [3,4]. This reported incidence has continued to grow exponentially and it is expected to rise to 1.08 million cases a year by the year 2030 [3,4]. The majority of OSCC patients are treated by an appropriate amalgamation of surgery, radiation, and chemotherapy [5]. Despite advances in treatment strategies, patients with OSCC suffer from a poor prognosis due to frequent recurrences and distant metastases [6]. Early detection and prevention are crucial factors in achieving better prognosis of OSCC with an improved 5-year survival [7].

OSCC has a multifactorial aetiology, including tobacco consumption, alcohol habits and viral (e.g., human papillomavirus) infections [8]. Field cancerization is the most accepted theory among researchers and clinicians, which explains frequent recurrences and metastases in OSCC [9]. Slaughter et al. [10], defined field cancerization as “pre-neoplastic mucosal area composed of epithelial cells with genetic or epigenetic changes beyond the original invasive malignancy, resulting in patients having several malignancies in various stages of development.” Potentially malignant lesions of the oral cavity include leukoplakia, oral submucous fibrosis, erythroplakia, and are characterised by white or red patches on the oral mucosa with epithelial histological changes ranging from hyperplasia to carcinoma in situ [11]. The malignant transformation rate of leukoplakia ranges from 0.13 to 34% [12] and the transformation rate for erythroplakia ranges from 1.1% to 40.8% [13]. Due to field cancerization, it is hypothesized that the entire oral mucosa will be exposed to the carcinogen, causing widespread premalignant and malignant changes [10]. This situation represents a dilemma to the surgeon and clinical cancer management team regarding the resection margins and treatment regimens, as complete resection of the tumour is essential for a good prognosis [14]. This emphasizes the need for an instrument that enables the evaluation of dysplastic lesions in-vivo, preventing the need for wide-spread preventive excision.

The inability to clearly define surgical margins intraoperatively in OSCC patients is the number one reason for the recurrence of primary tumours and leading to a debilitating recurrence and associated metastasis [15]. The current practices which help in determining surgical margins include visualisation, palpation, or frozen section histopathology [16]. Although frozen sections are accurate, they are associated with multiple drawbacks, including compromising the tissue integrity and being time consuming [17]. Avoiding unnecessary resection of healthy tissues is of utmost importance to the surgeons, due to the functional limitations post-operatively and severe impact on the quality of life of the patient [18,19]. This limitation also supports the requirement of an explicit and correct evaluation of the affected oral tissues preceding surgical resection.

An ideal solution to these problems would-be real-time histological evaluation by an instrument, which is non-invasive, time efficient, and sensitive enough to replace the gold standard of histopathology. This concept has been previously explored using narrow-band imaging [20,21], autofluorescence imaging [22,23], computed tomography [24], and confocal imaging [25]. The latter study was done with a handheld confocal laser endomicroscope comprising of a bundle fibre probe (Manua Kea Cellvizio) with IV fluorescein as the fluorescent dye. This technique allowed efficient visualization of the epithelial architecture with a fluorescent contrast on the intraoperative display [26]. A fluorescent contrast agent intensifies the contrast of cells, which are imaged with a blue laser. Confocal Laser Endomicroscopy, aka “*optical biopsy*”, is used to deliver the surgeon with real-time cellular resolution digital images (1 µm to a 1000-fold magnification) during surgical procedures, allowing effective analysis of the surgical margins and ensuring improved precision in the determination of tumour resection margins and preventing recurrence. This medical imaging modality enables an in vivo diagnosis and images can be acquired almost indefinitely whilst avoiding any iatrogenic harm to the patient.

Previously, CLE has shown effective imaging whilst diagnosing gastrointestinal neoplasia’s including Barrett’s oesophagus [27,28], intraepithelial neoplasia’s of the colorectal tract [29,30], pre-neoplastic and neoplastic lesions of the cervical epithelium [31,32], neoplastic lesions involving the bronchial epithelium [33,34,35], neoplastic lesions of the urothelial epithelium [36,37] and lesions of the brain or spinal cord [38,39,40]. The first report of CLE utility in the head and neck region, along with morphological associations with analogous H&E stained tissue sections, was described in 1999 [41], followed by multiple in vivo and in vitro studies [42,43,44,45,46,47,48,49,50].

To articulate comprehensive and conversant evidence-based recommendations for the coherent use of CLE, a systematic review and meta-analysis was designed, to assess the precision in the diagnosis of OSCC using histopathology as the reference standard.

## 2. Materials and Methods

A systematic review and meta-analysis was performed and the results were described according to the standard Preferred Reporting Items for Systematic Reviews and Meta-Analysis (PRISMA) statement [51]. The review protocol was registered in the PROSPERO International Prospective Register of Systematic Reviews (CRD42021278405).

### 2.1. Study Objective and Definition of Reference Standard

The search strategy followed the PICO (population, intervention, comparison, and outcome) guidelines [52]. The population comprised of OSCC patients; OSCC can be defined as squamous cell carcinomas in the oropharynx and oral cavity including tongue, palate, etc. The intervention used was the use of CLE for diagnosis of OSCC. True positives (TP), false positives (FP), true negatives (TN), and false negatives (FN) were the outcomes measured while detection of OSCC using histology were the reference standard. A diagnosis subsequent to histopathological analysis of a biopsy specimen (incisional or excisional) was considered. The study design had no limitations, as long as original data were specified. The main objective of this systematic review and meta-analysis was to assess the accuracy of CLE for the diagnosis of OSCC.

### 2.2. Literature Search Strategy

Block search was carefully chosen as the search strategy, as it adhered to the PICO approach. A reviewer (S.S.) explored the following databases till 30 July 2021: PubMed (keywords “(oral squamous cell carcinoma)” OR “(Oral Cancer)” AND “(confocal microscopy”), Web of Science (keywords “TS = (confocal microscopy AND oral squamous cell carcinoma or Oral Cancer) Timespan: All years. Indexes: SCI-EXPANDED, SSCI, A&HCI, CPCI-S, CPCI-SSH, BKCI-S, BKCI-SSH, ESCI, CCR-EXPANDED, IC.”) and Elsevier-SCOPUS (keywords “TITLE-ABS-KEY (“confocal microscopy” AND “squamous cell carcinoma” OR “(Oral Cancer)”). All references were extracted and duplicates were removed using the reference manager EndNote (version X9, 2020, Clarivate, Philadelphia, PA, USA).

To evaluate the applicability of studies, two reviewers (S.S. and X.J.) independently screened all retrieved articles by titles first followed by abstracts to determine their relevance. Possibly eligible articles were analysed after full-text recovery. Incongruities were discussed with a third reviewer (LJ).

### 2.3. Inclusion and Exclusion Criteria

The established eligibility criteria were: (1) the CLE instrument used in the study should be based on the principle of Fluorescent Laser Endomicroscopy only; (2) the examined lesions were OSCCs (well differentiated, moderately differentiated, and poorly differentiated histopathological subtypes); (3) histopathological diagnosis of OSCC was the reference standard, following the examination of the biopsy specimen (incisional or excisional); (4) only human studies were included; (5) no case reports or reviews were included; (6) articles written in English were exclusively included.

We excluded from the analysis: (1) all studies using Reflectance Confocal Microscopy; (2) all basal cell carcinomas or corneal/optical epithelial tumours; (3) all studies based on cytological smears; (4) no animal-based models or trials were included; (5) studies where full-text and recovery was unlikely, including a search of the relevant databases and attempting communication with the corresponding authors. Studies with overlapping populations found in studies were also excluded, including a single study with the most representative data. Moreover, the reference list of all the papers were reviewed to ascertain additional appropriate studies that may have been unnoticed whilst initial screening was undertaken.

### 2.4. Data Extraction

A reviewer (S.S.) extracted the data from the included studies, which was additionally validated by another reviewer (X.J.). The variables extracted included: the name of the first author, country, year of publication, tumour sites (percent frequency), number of reviewers validating the CLE device, fluorescent agent used, total patients and lesions, patient sex and age (mean/median, years), sensitivity of instrument, specificity of instrument, and confocal criteria employed for the diagnosis of OSCC.

### 2.5. Risk of Bias

Risk of bias and quality of included studies was evaluated using the Quality Assessment of Diagnostic Accuracy Studies) QUADAS-2 checklist for primary studies assessing the diagnostic accuracy in four KEY domains; Patient Selection, Index Test, Reference Standard, and Flow and Timing [53]. All four domains are appraised for risk of bias, and only the first three domains are appraised for applicability concerns. The risk of bias was assessed to be either high, low, or unclear on the basis of the following questions: (1) if the reference standard was more likely to correctly diagnose OSCC (2) if a threshold for index-test was respecified; (3); whether the patient selection was a sequential or random sample of patients enrolled; (4) if there was an appropriate interval between index test and reference standard.

### 2.6. Statistical Analysis and Meta-Analysis

Tables were created for each CLE-based diagnosis of OSCC with histopathology diagnosis of incisional or excisional biopsy specimens. The sensitivity, specificity, and corresponding 95% confidence intervals were represented using forest plots. Diagnostic accuracy in terms of sensitivity, specificity, and diagnostic odds ratio with 95% confidence intervals was evaluated on the basis of TP, FP, TN, and FN extracted from each of the included studies.

Sensitivity was estimated as the proportion of patients, correctly identified by CLE as having OSCC, and specificity as the proportion of patients, correctly identified on radiology as not having OSCC. The diagnostic odds ratio was defined as the odds of the CLE diagnosis being positive for a patient having OSCC relative to the odds of the CLE test being positive for patients not having OSCC. Meta-analysis of sensitivity and specificity were determined by designing a bivariate model (hierarchical logistic regression) [54] and the Hierarchal summary receiver operating characteristic curve (HSROC) was created. The HSROC curve represents sensitivity vs. specificity graphically and provides evidence concerning the general test performance across different thresholds. Within study and between studies variability was determined by this model.

Heterogeneity is calculated as Higgins I^2^, where I^2^ = 0% indicates no observed heterogeneity and I^2^ > 50% is categorised as substantial heterogeneity. Heterogeneity is a prominent attribute observed in almost all meta-analyses of diagnostic accuracy tests, which can be explained by variation in the index test efficiency due to varied suggestive diagnostic thresholds. Statistical analysis of the sources of heterogeneity was not performed as the size of the subgroups were insignificant (two or three studies per group).

Data management and statistical analyses was performed using the software packages STATA (v15.0; StataCorp LP, College Station, TX, USA), Review Manager (v5.3; Copenhagen, Denmark) and MetaDisc (v1.4; Madrid, Spain).

## 3. Results

### 3.1. Literature Search Results

**Figure 1:**
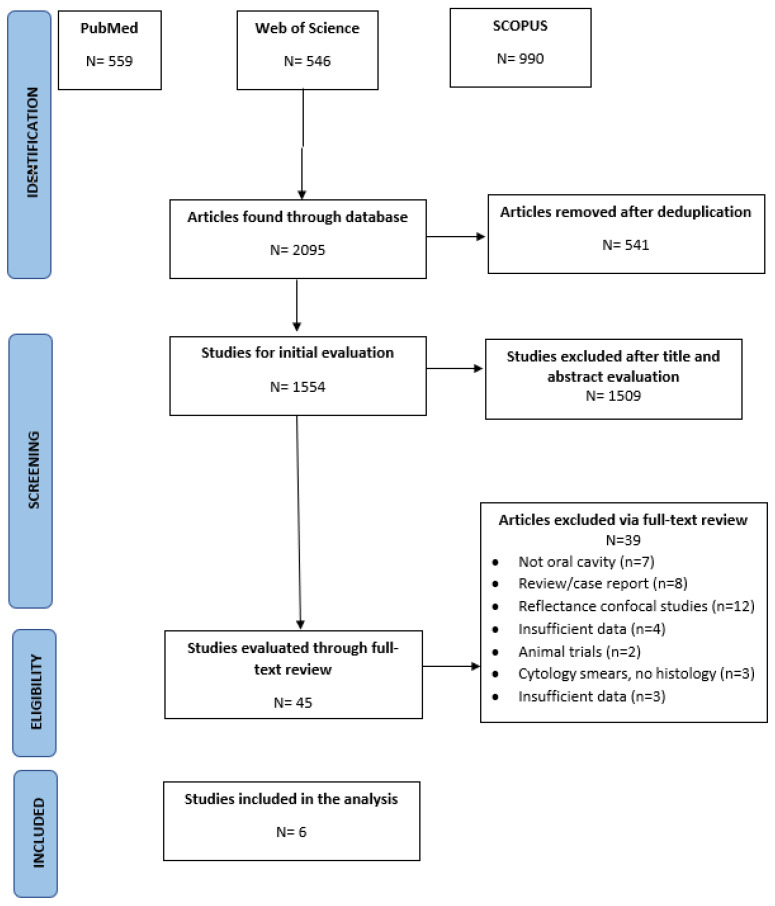
Screening process and results. Oral squamous cell carcinoma (OSCC).

The preliminary database search recognized a total number of 2095 of articles. After removal of duplicates, only 1554 articles persisted. After title and abstract assessment, 1509 articles were disqualified and 45 were selected for full-text recovery and further analysis. Thirty-nine articles were excluded after full-text analysis illustrated in Figure 1. Six studies totaling a number of 361 lesions in 213 patients were included in the final analysis [25,55,56,57,58,59]. The study characteristics are tabulated in Table 1.

Two out of the six included studies did not specify the patient details of mean age, number of males and females. The average percent of males and females across the remaining four studies were 62.2% and 37.8% respectively. The manufacturer of the CLE devices CellVizio, Vivascope 2500, FIGH-300S and CONVIVO was Mauna Kea Technologies (Paris, France), Lucid Inc. (Lucid Technologies, Henrietta, NY, USA), Fujikura or HDIG (Sumita) and Carl Zeiss AG (Oberkochen, Germany), respectively. The majority of studies were carried out in Germany. One study did not specify the site of OSCC in oral cavity [56]. Confocal criteria for OSCC diagnosis varied considerably between studies (Table 2).

The included studies exhibited low or unclear risk for bias and applicability concerns in all domains. One study (16.66%) had an unclear (*n* = 1) risk of bias regarding patient selection with an indeterminate patient selection procedure. Five studies completely described the patient selection protocol. One study presented high and uncertain applicability concerns due to restrictions applied to the studied population (including lesions of suspected premalignancy and malignancy) and inclusion of the contralateral oral mucosa of patients, which overlooks the concept of field cancerization and applicability of the tool.

Two out of the six included studies had a high risk of bias or unclear risk of bias concerning the index test being mostly attributed to the blinding of investigators to patient characteristics. Most of the studies had low applicability concerns in the index test area due to clear demarcation of malignant changes in the observed epithelial cells.

Five of the included studies had a low risk of bias concerning the use of the reference standard due to clearly defined histopathological and confocal criteria, only one study had no clear diagnostic criteria and were at high risk of bias due to inadequate referencing standards. With respect to applicability concerns of the reference standard, only one study had a high risk due to the reference standard determined by an expert clinical diagnosis, whilst one study did not specify the assessors’ experience level.

According to the QUADAS-2 tool, in the domain of flow and timing, three studies had a high risk of bias as these studies comprised a fair number of all benign and suspected dysplastic lesions.

### 3.2. Diagnostic Accuracy of CLE and Meta-Analysis

Whilst there are limitations to the conclusions that be drawn from a meta-analysis, the meta-analysis does give a general overview of the calculated sensitivity and specificity of any confocal microscope in different situations. Although, the studies used dissimilar tools in different settings, the actual point estimates of sensitivity and specificity depict ‘threshold effect’ in diagnostic clinical research.

**Figure 2:**
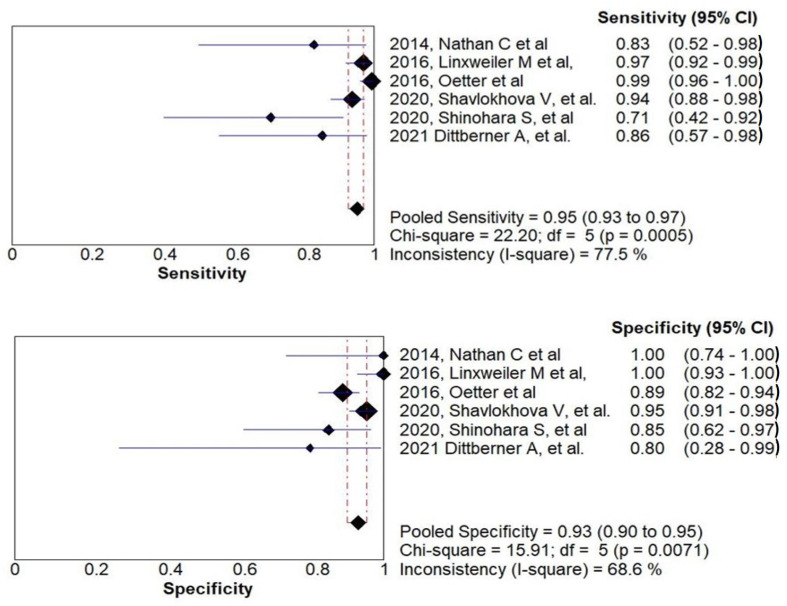
Forest plots for each specific study and their pooled estimates of sensitivity and specificity with their heterogeneity statistics of confocal laser endomicroscopy for the diagnosis of oral squamous cell carcinoma.

All six studies were included in the meta-analysis. The results of the meta-analysis are reported but with its limitations, and attention against variation and potential biases. Sensitivity ranged from 71.4% to 99.3% and specificity ranged from 80% to 100%. The analysis revealed a pooled sensitivity and specificity of 95% (95% CI, 92.9–97%; I^2^ = 77.5%) and 93% (95% CI, 90–95%; I^2^ = 68.6%). The plot of CLE sensitivity and specificity with the summary values in the diagnosis of OSCC in the involved studies is illustrated in Figure 2.

The positive likelihood ratio ranged from 4.28 (95% CI, 0.73–25.06) to 98.5 (95% CI, 6.24–1554.1) and the negative likelihood ratio extended from 0.008 (95% CI, 0.001–0.055) to 0.3 (95% CI, 0.14–0.78). The pooled positive likelihood ratio and negative likelihood ratios were 10.85 (95% CI, 5.4–21.7; I2 = 55.9%) and 0.08 (95% CI, 0.03–0.2; I2 = 83.5%). The estimated diagnostic odds ratio (DOR) ranged from 14.1 (95% CI, 2.61–76.6) to 2861.7 (95% CI, 151.3–54,123.7). The pooled DOR was 174.45 (95% CI, 34.51–881.69; I2 = 73.6%).

**Figure 3:**
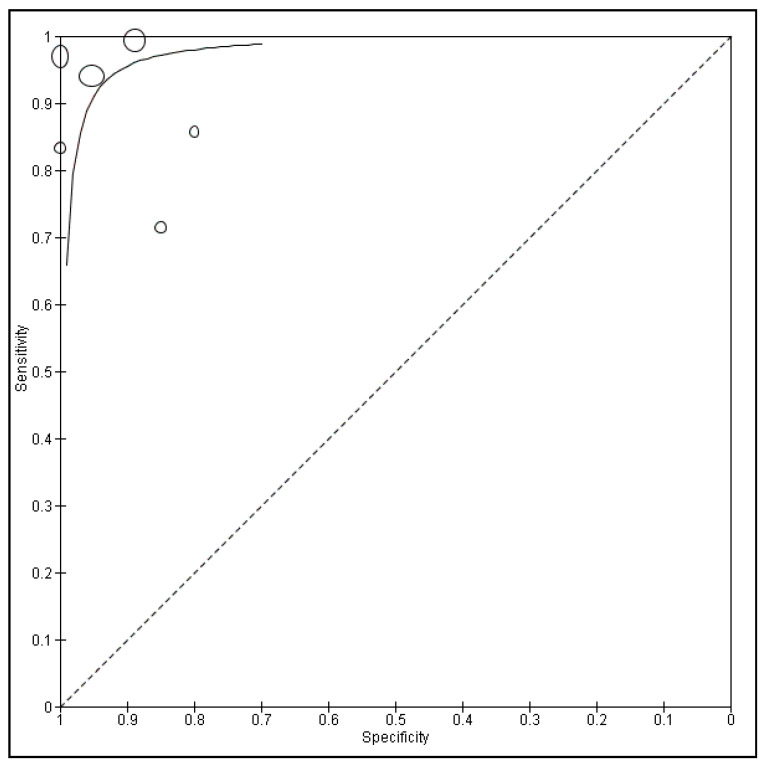
HSROC (hierarchal summary receiver operating characteristic) curve of sensitivity (Y-axis) vs. specificity (X-axis) of CLE for diagnosing OSCC. Points characterize the sensitivity and specificity of a single study. The sample size of the study was proportional to the size of the point. The solid line displays the summary ROC curve.

The form of the HSROC curve in Figure 3 and the estimated area under the curve (AUC) was 0.97, which suggested the absence of a threshold effect. The summary ROC curve in Figure 3 describes that effect, which explains most of the heterogeneity in these studies. Additionally, the random-effects method considered the variation between studies. The shape of the prediction region is a graphic illustration of the amount of heterogeneity between studies. It is reliant on the conjecture that the data follows a normal distribution and should therefore not be over-interpreted.

### 3.3. Heterogeneity Analysis

With reference to heterogeneity analysis, a Spearman correlation coefficient of 0.314 (*p* = 0.544) was calculated which proposed the lack of a threshold effect. Statistical analysis of the sources of heterogeneity was not performed as subgroups were too small (two or three studies per group).

The foundations of bias include variation in (i) sites within the oral cavity assessed; (ii) type of CLE device; and (iii) level of CLE training of the reviewers.

As per the general methodology rules specified for Cochrane reviews [60], “tests for funnel plot asymmetry (to assess publication bias) should be used only when there are at least 10 studies included in the meta-analysis, because when there are fewer studies the power of the tests is too low to distinguish chance from real asymmetry.” As six studies have been included in the meta-analysis, a funnel plot was not performed.

**Figure 4:**
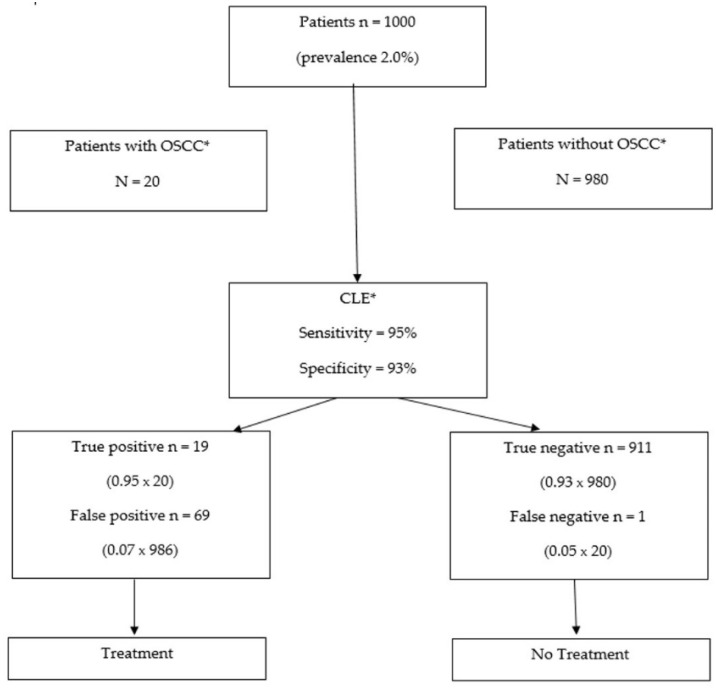
The use of CLE for OSCC diagnosis in a cohort of 1000 subjects. CLE would lead to 88 patients being treated, of which 69 would not need to be treated; 911 patients would not receive treatment, of which only one would have demanded treatment. * Abbreviations: OSCC—Oral Squamous Cell Carcinoma; CLE—Confocal Laser Endomicroscopy.

Based on latest epidemiological data [4], the global prevalence of OSCC is 2%. Using existing data along with the results of this study, the total number of true positives, false positives, true negatives, and false positives can be projected in a hypothetical cohort of 1000 patients. This means that 20 patients in this cohort would have OSCC. If we use CLE as a diagnostic tool for OSCC (sensitivity of 95% and a specificity of 93%), one of these 20 OSCCs would go undetected, while 69 patients would be needlessly treated (Figure 4).

## 4. Discussion

CLE is a potentially useful diagnostic tool that is non-invasive and allows real-time cellular imaging of the epithelium of the upper layers of the epithelium at resolutions comparable to histology. The criteria for CLE diagnosis of OSCC are easy to learn and even non-experts in the field of CLE have been able to make a precise diagnosis of OSCC by using these criteria [56]. Previous research has indicated the efficient and precise ability of CLE to envisage dysplastic head and neck squamous cell mucosa, with close reproducibility of the histopathological diagnosis [61,62].

This systematic review and meta-analysis compares histopathological diagnosis from in/ex vivo specimens to the diagnostic precision of CLE by means of analysing the results of 6 studies which comprised a total of 361 lesions. The search strategy used wide-ranging keywords in various relevant databases to find as many studies as possible.

The outcomes of the meta-analysis indicate a sensitivity of 95% and a specificity of 93% when using CLE for diagnosis of OSCC. However, care must be taken while interpreting these extraordinary values of both sensitivity and specificity. The substantial heterogeneity indicates the direct assessment of the diagnostic accuracy of CLE amongst the included studies improbable. CLE sensitivity for the diagnosis of OSCC ranged between 71.4% to 99.3%, and its specificity ranged between 80% and 100%. Though statistically insignificant (most likely due to inadequate statistical power), the discrepancies could still be explained by the diverse confocal criteria and dissimilar experimental designs (in vivo vs. ex vivo), but also investigator skill, and most likely other indefinite heterogeneity sources. Even when utilising similar diagnostic criteria, the experience of an investigator could have an influence on the diagnostic accuracy as it has been demonstrated that there is a strong correlation of expertise level and the interpretation accuracy [57].

Of the six studies included in our review, three are ex vivo [55,57,58] in design and three are in-vivo [25,56,59] in design. However, when comparing the CLE images for fresh-frozen and formalin-fixed tissue from the patients, only marginal differences (regarding the range of brightness) in morphology were observed, with no variation in the resolution of the images. Thus, it can be stipulated that the images on ex vivo specimens would be largely reproducible in an in vivo situation [55].

### 4.1. Clinical Relevance

The efficiency and acceptability of this instrument has been addressed in the study by Nathan C et al. [25], in which they explain the advantage of CLE imaging as it decreases the sampling errors encountered during tissue biopsy or could lead to the decision of avoiding a biopsy altogether and leading to real-time management decisions.

The gold standard treatment for oral dysplastic lesions is excision or laser ablation, and lower grade suspicious lesions are usually kept under observation, due to possible chances of regression (9–45%) [63]. However, this decision is highly criticised due to higher rates of recurrence, metastasis, and incipient malignant progressions, and it is argued that clinical examinations and palpations are insufficient in determining the malignant potential of a visually low suspicious presentation of a lesion [64]. Hence, there is the potential utilization of CLE as a surveillance tool, which could aid in diagnosing which lesions can be observed instead of being resected and which lesions demand enhanced management.

In vivo CLE could develop into a very valuable and beneficial instrument in the diagnosis of OSCC; but, to be regarded as an adjunct to the gold standard reference of histopathology, this non-invasive technique ought to have the capacity to distinguish between the histopathological grades of OSCC [65]. Although the treatment strategies for OSCC are largely based on the TNM staging of the tumour [66], the histopathological grade is also of critical importance when strategizing the therapeutic approach to treat an OSCC patient.

### 4.2. Strengths and Limitations

The included studies had some limitations such as the improper visualization of the dorsal surface of the tongue due to the keratinized filiform papillae and the limited accuracy of detection of lesions below the superficial mucosa [25,55]. Most of the included studies had a small sample size and the reproducibility of the results are not reliable enough to make concrete diagnosis and treatment decisions [25,58,59]. Due to ex vivo experiment design, one study is limited by the unavailability of fresh-frozen tissues for all included patients [55] as this limits the reciprocation of their results. This particular study highlights the accuracy of a non-invasive sensitive imaging modality which can be used as a screening instrument. Early detection of OSCC can greatly improve the prognosis and thus help in reducing the current burden. Although it is still not a verified replacement of histopathology, its non-invasive nature does ensure a large screening based program, with early detections and treatments. However, we would like to acknowledge the fact that precancerous lesions were not recruited or considered for inclusion in this study and there is no evidence to support the fact that it may be successful to evaluate precancerous lesions. Given the accuracy and highly sensitive reproduction of cellular details, it can only be speculated that it will reproduce similar results for precancerous lesions and can be used for screening purposes.

One of the included studies notes that no study so far has investigated the clinical and surgical utility of CLE, especially intraoperative visualization of distinct cancerous and non-cancerous tissue margins, to limit the extent of surgical invasions [55].

To enable homogeneity, future studies could consider reporting the number of lesions analyzed, the skilled experience of the investigator, and attendance of skill development courses. Development and validation of a standardized confocal criteria for OSCC diagnosis via international agreement is desirable. Global consensus is essential for this instrument to begin its journey towards replacing the invasive surgical and histopathological techniques for screening purposes

### 4.3. Future Directions

Regarding the future scope of CLE, transference of the first tentative results of CLE in the human oral cavity into an effective and evidence based clinical setting will be a crucial step. Recent research has shown an evolution of artificial intelligence algorithms and the utilization of computational methods for an accurate diagnosis and prognosis of head and neck cancers [67]. Artificial intelligence science along with precision-based optical imaging systems such as confocal microscopy greatly improve the prospects of improving screening and prognostic outcomes of OSCC.

Further studies exploring the diagnostic accuracy of in vivo confocal laser endomicroscopy for OSCC are expected in future. To help comparability of the results it is recommended that the histopathological assessment of the excisional biopsy specimen be utilized as a reference standard.

## 5. Conclusions

Confocal laser endomicroscopy is a technique that may assist in the diagnosis of oral squamous cell carcinoma. A decisive conclusion relies on an increased number of studies investigating this technique that follow a homogeneous methodological approach, which will allow for a comparable assessment.

## Figures and Tables

**Table 1:** Characteristics of included studies.

Year	Author	Country	Site Distribution	Examination Setting	Ni. of Reviewers	CLE * Device	Fluorescent Agent Used	Total Patients	Total Sites	Patient Gender (%) and Age (Mean/Median)	Reference
2021	Dittberner A, et al.	Germany	Oropharynx (52.9%), oral cavity (35.3%), and hypopharynx (11.8%).	In vivo	2	CONVIVO, Carl Zeiss AG, Oberkochen, Germany	Fluorescein	13	30	Mean age—1.9 years M—69% F—31%	Conventional histopathology
2020	Shinohara S, et al.	Japan	Hypopharynx (30%), larynx (10%) lower gingiva (20%), tongue (20%), oropharynx (20%)	Ex vivo	NS*	FIGH-300S or FIGH 350S, Fujikura or HDIG, Sumita	Acriflavine	10	10	Mean age—67.7 years M—80% F—20%	Conventional histopathology
2020	Shavlokhova V, et al.	Germany	Lip (7%), palate (18%), tongue (37%), buccal mucosa (15%), floor of the mouth (23%)	Ex vivo	3	Vivascope 2500 Multilaser, Lucid Inc., Rochester, NY*, USA*	Acridine Orange	70	70	Mean age—68.7 years M—52.2% F—47.8%	Conventional histopathology
2016	Oetter et al.	Germany	NS	In vivo	6	Cellvizio, Mauna Kea Technologies, Paris, France	Fluorescein Alcon	NS	95	NS	Conventional histopathology
2016	Linxweiler M et al.	Germany	Tonsil cancer (26%), tongue base cancer (24%), hypopharyngeal cancer (15%), tongue cancer (10%), cancer of the soft palate (8%), cancer of the pharyngeal wall (7%), cancer of the floor of the mouth (6%), cancer of the buccal mucosa (3%)	Ex vivo	12	Cellvizio system (Mauna Kea Technologies, Paris, France	Acriflavine hydrochloride	99	185	NS	Conventional histopathology
2014	Nathan C et al.	USA *	Tongue (66.6%), tonsil (4.7%), vocal cord (14.2%), epiglottis (4.7%), floor of mouth (4.7%), retromolar triangle (4.7%)	In vivo	4	CellVizio; Mauna Kea Technologies, Paris, France	Fluorescein Alcon	21	21	Mean Age—64.2 years M—47.6% F—52.3%	Conventional histopathology

* Abbreviations: CLE—Confocal Laser Endomicroscopy, M—Males, F—Females, NS—Not Specified, NY—New York, USA—United States of America.

**Table 2:** Criteria utilised for diagnosis of oral squamous cell carcinoma by the studies included in this review.

Author, Year, [Reference]	Laser Confocal Endoscopy Microscopic Criteria
Dittberner, A, 2021 [59]	Chronic inflammation, dysplasia-free normal tissue, none to severe artefact classification, tissue architecture, cell morphology, fluorescence leakage, and the vessels.
Shinohara S, et al., 2020 [58]	Uniformity of nuclear size and shapes, cell density, nuclei and cytoplasm of cells
Shavlokhova V, 2021 [57]	Disturbed polarity of the basal cells, basal cell hyperplasia, irregular epithelial stratification or disturbed maturational sequence, cellular pleomorphism/anisocytosis, nuclear hyperchromatism, prominent nucleoli, intraepithelial keratinization, increase in nuclear cytoplasmic ratio
Oetter et al., 2016 [56]	Homogeneity, intercellular gaps, cell morphology, fluorescein leakage, vessel morphology
Linxweiler M et al., 2019 [55]	Variable cellular morphology, lack of cytoplasmic membranes, and a hazy, moth-eaten appearance.
Nathan C et al., 2014 [25]	Normal or non-dysplasia, dysplasia, or cancer.

Risk of bias and quality assessment of study reports. The outcome of the methodological quality assessment of the included studies is explained in Table 3.

**Table 3:** Methodological assessments using QUADAS-2 tool. Each domain is assessed for risk of bias and first three for their applicability concerns.

Studies	Domain 1 Patient Selection		Domain 2 Index Test(s)		Domain 3 Reference Standard		Domain 4 Flow & Timing	Total Score
	Risk of Bias	Applicability Concerns	Risk of Bias	Applicability Concerns	Risk of Bias	Applicability Concerns	Risk of Bias	
Dittberner A, et al.	Low	Low	Low	Low	Low	Low	Low	0
Shinohara S, et al	Low	Unclear	High	Low	High	High	Low	7
Shavlokhova V, et al	Low	Low	Low	Unclear	Low	Low	High	3
Oetter N, et al	Unclear	Low	Low	Low	Low	Low	High	3
Linxweiler M, et al	Low	High	Unclear	Low	Low	Low	Low	3
Nathan C, et al	Low	Low	Low	Unclear	Low	Unclear	High	4

(Low—low risk (0 points), high—high risk (2 points) or unclear—unclear risk (1 point)).

## Data Availability

This study did not report any data.
